# Hysterosalpingography in the workup of female infertility: indications, technique and diagnostic findings

**DOI:** 10.1007/s13244-012-0183-y

**Published:** 2012-07-17

**Authors:** Adrian C. Schankath, Nikola Fasching, Cornelia Urech-Ruh, Michael K. Hohl, Rahel A. Kubik-Huch

**Affiliations:** 1 Institute of Radiology, Kantonsspital Baden AG, 5404 Baden, Switzerland; 2 Department of Gynaecology and Obstetrics, Kantonsspital Baden AG, 5404 Baden, Switzerland

**Keywords:** Infertility, Female, Hysterosalpingography, Fallopian tubes, Pregnancy

## Abstract

**Objectives:**

To evaluate the spectrum of diagnostic findings in hysterosalpingography (HSG) examinations performed at our institution between 2006–2010 and their prognostic significance for treatment decisions and fertility outcomes.

**Methods:**

Patients were filtered from our PACS. Pathological HSG studies were re-evaluated. Indications for referral, technical success and diagnostic findings were analysed. Pathological findings were correlated with further diagnostic workups, treatments and fertility outcomes.

**Results:**

Of 411 HSG examinations, 226 (55 %) were normal, 94 (23 %) showed minor abnormalities and 5 (1.2 %) were not diagnostic. Eighty-six (21 %) examinations were pathological. Twenty-nine patients underwent subsequent laparoscopy, during which proximal tubal occlusion diagnosed at HSG was ruled out in 9/23 cases. Follow-up information was unavailable for 20 patients. Nineteen of 66 patients with follow-ups after pathological HSG had at least one subsequent successful pregnancy. Forty-one patients had no further treatment and no pregnancies.

**Conclusions:**

The detection rate for pathologies at HSG was low (21 %). There was a high false-positive rate (39 %) for proximal tubal occlusion, most likely because of spasms, demonstrating the importance of delayed imaging after injection of antiperistaltic agents. HSG remains a valuable diagnostic tool. Our results, however, indicate that this technique should be more selectively indicated.

***Main Messages*:**

• *Good acceptance of HSG by the patients. No complications with antibiotic prophylaxis.*

• *Low detection rate (21 % pathological exams) for pathologies in our study.*

• *High false-positive rate for proximal tubal occlusion.*

• *This demonstrates the importance of waiting longer after injection of buscopan.*

• *High pregnancy rate in pathological cases: Indication too broad or even a therapeutic effect of HSG?*

## Introduction

Approximately 15 % of couples are affected by infertility, which is defined as the inability to conceive after 12 months of regular unprotected sexual intercourse [1]. Common causes of infertility include male factor (45 %), ovulation disorders (37 %) and tubal damage (18 %) [2]. A combination of several factors is found in approximately 20 % of all couples. The etiology of tubal damage can be intrinsic (ascending salpingitis, including salpingitis isthmica nodosa) or extrinsic (peritonitis, endometriosis and pelvic surgery). The most common causes of pelvic inflammatory disease (PID) are *Chlamydia trachomatis*, *Neisseria gonorrhoeae* and multibacterial infections [3]. Studies have demonstrated that the severity of tubal damage found in infertile women is directly related to their serum chlamydia antibody IgG titer (CAT) [4].

Uterine cavity abnormalities can be a contributing cause of subfertility in 10 % of women. Abnormal uterine findings are reported in as many as 50 % of women with recurrent implantation failure [5]. These findings include endometrial polyps or fibroids, which are observed as filling defects or uterine wall irregularities using hysterosalpingography (HSG). HSG can also demonstrate intrauterine adhesions and congenital abnormalities [1].

Imaging plays a key role in the diagnostic evaluation of female infertility [6]. Transvaginal ultrasound (TVUS) is a standard, first-choice procedure. Abnormal findings can be further evaluated with saline or contrast hysterosalpingo sonography [1]. Hysterosalpingo contrast sonography (HyCoSy) has been found to be highly sensitive, specific and accurate in identifying uterine abnormalities, e.g., polyps. However, it is of limited value for the assessment of tubal abnormalities. Magnetic resonance imaging (MRI) can be used to evaluate congenital Müllerian duct anomalies and to diagnose adenomyosis, leiomyoma and endometriosis; however, its role in tubal assessment is presently limited [7, 8].

The primary role of HSG is to evaluate the morphology and the patency of the fallopian tubes. The fallopian tubes should appear as thin, smooth lines that widen in the ampullary portion. Tubal abnormalities observed with HSG can be congenital, or due to spasm, occlusion or infection. Tubal occlusion manifests as an abrupt cutoff of contrast material with non-opacification of the distal fallopian tube, and can be unilateral or bilateral. Peritubal adhesions prevent contrast material from spilling into the abdominal cavity and distributing freely [8, 9].

HSG can also be helpful in evaluating uterine cavity abnormalities. It is considered to have a high sensitivity (60–98 %) but low specificity (15–80 %) in detecting uterine abnormalities, and hysteroscopy remains the method of choice for the final assessment. The differential diagnosis of intrauterine filling defects by HSG includes polyps, endometrial hyperplasia, submucosal fibroids, intrauterine adhesions and septa. These findings necessitate further investigation with hysteroscopy to confirm and possibly treat the pathology [1]. In the literature, the results of HSG studies have been shown to be important for selecting patients for diagnostic laparoscopy with chromopertubation [9, 10].

In 2008, guidelines published by the European Society of Human Reproduction and Embryology (ESHRE) recommended that a semen analysis and an ovulation assessment should be performed before a tubal patency test. Women with a high probability of pathological conditions should be offered a first line laparoscopy. This approach has the advantage that any tubal or pelvic pathology can be investigated and treated at the same time. The ovaries can be assessed by TVUS according to the ESHRE guidelines [11, 12]. Following these guidelines, we advise women to undergo a first line laparoscopy combined with hysteroscopy if they have a history of previous pelvic surgery, PID, elevated CAT, or severe dysmenorrhea or dyspareunia.

The purposes of this study were to evaluate all pathologies that were diagnosed by HSG in the workup of female infertility at our institution between 2006 and 2010, to describe the spectrum of diagnostic findings, and to correlate the diagnostic findings with clinical findings and outcomes.

## Patients and methods

Patients were identified by searching the radiology information system (RIS) for all HSG examinations from September 2006 (introduction of our Picture Archiving and Communication System; PACS) to April 2010. A total of 411 HSG examinations were identified and included in the study. The ages of the patients ranged between 22 and 42 years, and the mean age was 32.6 years.

For most patients, diagnostics and treatment were performed in our department. Some patients were sent to our department only for tubal assessment by their gynaecologists or family doctors. In selected cases, follow-up information concerning pregnancy or pathological findings was therefore not available.

Our department of gynecology specialises in the diagnosis of infertility and its treatment, including in vitro fertilisation (IVF). The Department of Gynecology as well as the Institute of Radiology are certified by DIN EN ISO 9001:2000. Approximately 450 intrauterine insemination (IUI) cycles and 450 fresh/frozen IVF/intracytoplasmatic sperm injection (ICSI) cycles were performed.

### HSG examination

Patients were scheduled between the 7th and 12th day of their menstrual cycle to ensure a thin endometrium, which makes interpretation of the images easier. A preovulatory exam was performed to exclude an early pregnancy. Contraindications, beside pregnancy, included pelvic inflammatory disease and severe allergy against iodine contrast agents. The patients were empty-stomached for the procedure.

At our institution, HSG is performed under sedation with propofol. This sedation is performed by an anesthiologist, and the patient is preinformed of the theoretical risks, e.g., aspiration or allergic reaction. Therefore, the HSG procedure is very well tolerated by the patients. An antibiotic is administered if tubal occlusion has been diagnosed to prevent pelvic infection. Furthermore, antibiotic prophylaxis is also given to patients at risk for infection, e.g., those with cardiac valvular disease.

The cervical os is cannulated by the gynaecologists with a Cohen catheter (manufactured by Storz, Germany). When the catheter is in place, water-soluble iodinated contrast medium (Telebrix® Hystero Guerbet AG, France) is applied under fluoroscopic control. The contrast medium demonstrates the morphology and contours of the uterine cavity. Further injection of contrast medium will outline the cornua, isthmic and ampullary portions of the tubes, and will show the degree of spillage into the abdominal cavity and the patency of the tubes. If one or both tubes show no contrast spillage into the abdominal cavity, the possibility of tubal spasm must be excluded by intravenous administration of scopalamin (Buscopan® 20 mg) and further contrast administration. As soon as the patient's heart rate—as sign of the effect of Buscopan®—shows an increase on the monitoring system, additional fluoroscopic imaging is performed.

At our institution, four radiographs are taken after contrast administration in each patient, including images of the early and complete filling phases of the uterine cavity, outlining of the fallopian tubes and the contrast spillage into the abdominal cavity. Additional images can be recorded in selected cases, e.g., following administration of antiperistaltic agents, but are rarely necessary. For radiation protection, our medical staff is continuously trained to reduce the radiation exposure to a minimum. The radiation dose was calculated for random samples. The values varied between 1.5 and 3 mSv (in comparison, the mean radiation dose of a chest X-ray is 0.01 mSv). The dose was dependent on the fluoroscopy duration and patient-specific factors. Higher doses in some individual cases can occur. Following the examination, the radiologist and gynaecologist evaluated the images together, and a diagnosis was made in consensus. The radiological report and images were archived in our PACS.

### Data analysis

All reports on the 411 selected patients were reviewed for this study by two radiologists (AS, RK) together with a gynaecologist (NF). In all cases in which pathology was described in the report, the images were re-evaluated. The size and morphology of the uterine cavity were assessed. This included searching for uterine filling defects, contour abnormalities of the cavity as well as abnormal positioning. The images were reviewed to determine if the fallopian tubes were existent and patent.

**Fig. 1:**
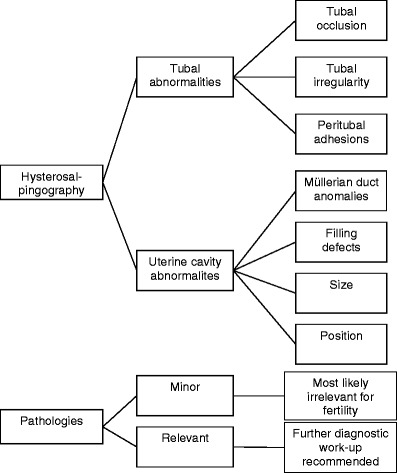
Scheme used for the characterisation of HSG findings, adapted from [6]

Tubal pathologies were categorised by occlusion (one- or two-sided, proximal and distal, postsurgical, spasm), tubal irregularity (postinfectious) and peritubal adhesions (e.g., PID). The degree and distribution of contrast spillage were recorded. Abnormalities of the uterine cavity were categorised into Müllerian duct anomalies (e.g., uterus bicornis) and filling defects (myoma, scars/adhesions). The size and position of the cavity were assessed. Pathologies were categorised using the diagram shown in Fig. 1 [6].

### Correlation of clinical findings with fertility outcomes

Sonographic, hysteroscopic and laparoscopic findings, and further information (i.e., history of previous surgery or infection) were retrieved from the gynaecology case histories for all patients with a pathological HSG. Indications for referral, technical success and diagnostic findings were analysed. Pathological findings were correlated with further diagnostic workup, fertility treatment and fertility outcome (i.e., pregnancy).

## Results

No immediate, i.e., allergy to the iodinated contrast agent or propfol, or delayed, i.e., pelvic infections, complications were observed during or following any of the 411 examinations. Of the 411 HSG examinations, 226 (55 %) were normal. In 15 of the examinations that were assessed as normal, administration of Buscopan® was required to differentiate spastic tubal occlusion from true tubal occlusion. There were minor abnormalities observed in 94 examinations (23 %). Five examinations (1.2 %) were not diagnostic (early termination of the exam due to venous filling of the uterine plexus).

In 86 (21 %) of the examinations, at least one pathology was described that was probably or possibly relevant to female infertility (Table 1). The detailed analysis of our study was based on this subpopulation. There was a statistically significant age difference between patients with normal (mean 31.8 years) and pathological (mean 34.1 years) HSG examinations (P-value = 0.0001).

**Table 1:** Summary of the distribution of pathologies and fertility outcomes, i.e., pregnancies and abortions following the 86 HSG examinations that revealed pathological findings

Diagnosis at HSG:	Results of further workup:	Outcome:
Uterine pathologies (30):		
Mucosal irregularities (4)	Mucosal irregularities (4)	3/4 patients pregnant
Filling defects of the uterine cavity (15)	Normal TVUS (4)	1/4 patients pregnant
	Endometrial polyp (1)	Pregnancy after resection
	Uterus myomatosus (1)	No pregnancy
	Intramural and subserous myomas (1)	No pregnancy
	Invasive endometriosis and scarring (1)	One miscarriage
	Filling defects with unavailable follow-up information (5)	Unknown
	No workup, insemination therapy (2)	No pregnancies
Müllerian duct anomalies:		
Arcuate uterus (5)	No workup (4), normal HSC and LSC apart from endometriosis (1)	1 normal pregnancy, 1 abortion
Hypoplastic uterus (4)	2 with normal ultrasound/HSC	No pregnancies
Uterus septus (1)	Confirmation at HSC, septum resected, no further follow-up information	Unknown
Uterus bicornis bicollis (1)	Planned laparoscopy	Still in therapy
Tubal pathologies (47)		
One-sided proximal tubal occlusion (17)	History of salpingectomy (5)	No pregnancies (4), unknown (1)
	Normal tubal morphology at laparoscopy (4)	2 patients with successful pregnancies, 2 with miscarriages
	Hydatid cyst occluding tube at laparoscopy (1)	No pregnancy
	History of sterilization and refertilization (1)	No pregnancy
	One-sided tubal malformation (1)	Spontaneous pregnancy
	No further workup (2)	No pregnancies
	No follow-up information available (3)	Unknown
Two-sided tubal occlusion (6)	Normal tubal aspect at laparoscopy (3)	1 miscarriage after insemination
	Normal tubal aspect, endometriosis (1)	No pregnancy
	Two-sided sactosalpinx (1)	No pregnancy
	Tubes patent at laparoscopy, additional myoma identified and resected (1)	1 miscarriage after spontaneous pregnancy
Diagnosis at HSG:	Further workup results:	Outcome:
Tubal pathologies (continued)		
Sactosalpinx (5)	Unknown (1), therapeutic fimbriostomy (3), one of these tested positive for chlamydia	No pregnancies
	Spontaneous pregnancy before workup (1)	1 spontaneous pregnancy
Postinfectious tubal irregularities (2)	No further workup	1 spontaneous pregnancy
Postoperative tubal pathology (1)	No further workup	No desire for children
Left tube occluded, right salpingitis isthimica nodosa (1)	Left tube was resected, right normal aspect at LSC	No desire for children
Right tube occluded, both tubes with postinfectious abnormalities (1)	LSC planned	2 spontaneous abortions years ago
Filling defect in a proximal tube (1)	Unknown	
Salpingitis isthimica nodosa (1)	Unknown	
Aspect of peritubal adhesions (12)	Additional polyp identified at HSC, normal tubes at LSC (1)	1 miscarriage (patient with resected polyp)
	No further consequence (10)	1 spontaneous pregnancy
	Unknown (1)	2 pregnancies after ICSI
Combined uterine and tubal pathologies (9)		
Filling defects and one-sided proximal tubal occlusion (3)	Tubes: Normal tube morphology at LSC but not patent (2), history of salpingectomy (1). Uterus: Polyps (1), normal morphology (1), unknown (1)	No pregnancies
Filling defect and sactosalpinx (1)	No follow-up	Unknown
Sactosalpinx and cervical pathology (1)	Salpingitis follicularis, history of extrauterine gravidity	No pregnancy
Filling defect and adhesions (1)	Polyp removed at HSC	No pregnancy
Tubal both-sided occlusion, arcuate uterus	No workup	Unknown
One-sided tubal occlusion, synechiae (1)	Proximal tube resection and anastomosis. Afterwards no further contact with patient	Unknown
Tubal irregularities (possibly postinfectious), minor filling defects (1)	Normal TVUS	1 sponataneous abortion and 1 normal pregnancy following

**Fig. 2:**
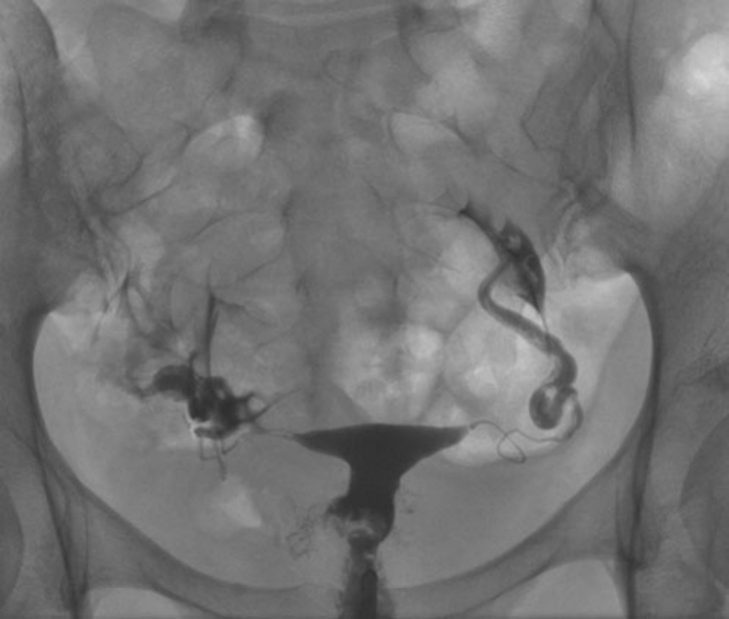
A 35-year-old patient: The irregular-shaped defect localised at the right side of the cervix was identified as a myoma using transvaginal sonography. No tubal pathology was performed<query ID="Q1"><query_paragraph>Should this be: "No tubal pathology test was performed"? Please check.</query_paragraph></query>. No follow-up information was available for this external patient

**Fig. 3:**
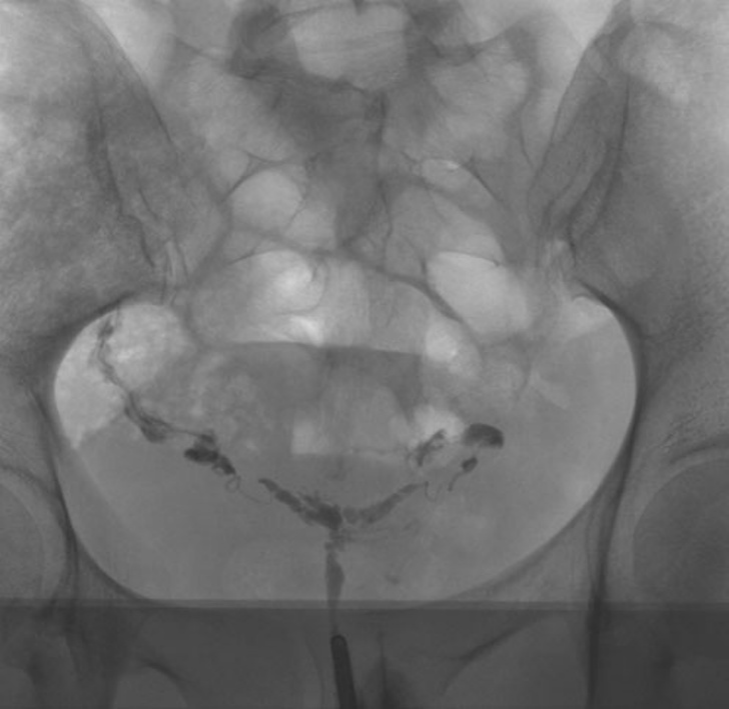
A 36-year-old patient: Small and irregular bordered uterine cavity with multiple linear constrictions. LSC and HSC were performed, and the diagnoses of invasive endometriosis with multiple scars and a single submucosal myoma were established. The submucous myoma, which was not visible in HSG, was resected. Subsequently, the patient had two spontaneous pregnancies; the first resulted in a spontaneous abortion, and the second was successful

**Fig. 4:**
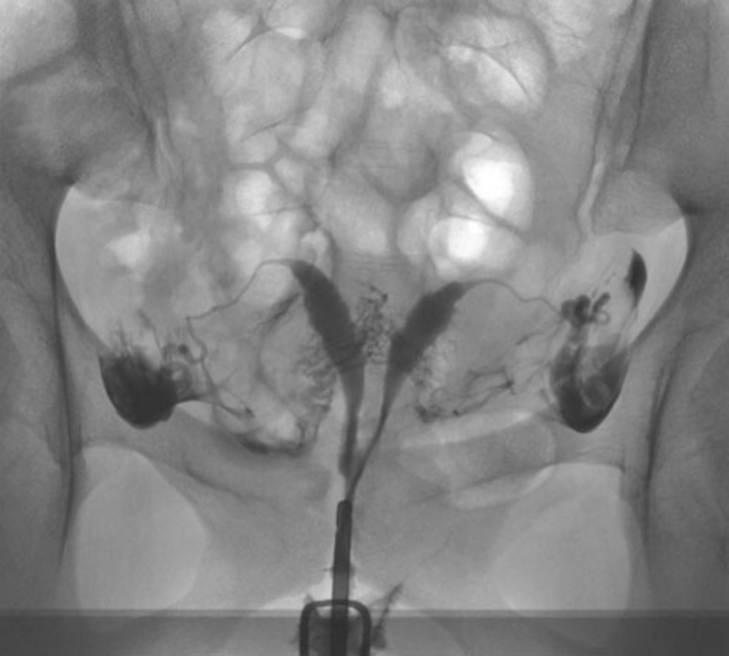
A 32-year-old patient: Symmetrically separated uterine cavity (uterus septus). Also, notice venous intravasation of the contrast medium (this can impede the image interpretation). LSC and HSC were performed, and the septum was resected. After this procedure, the patient was lost to follow-up

**Fig. 5:**
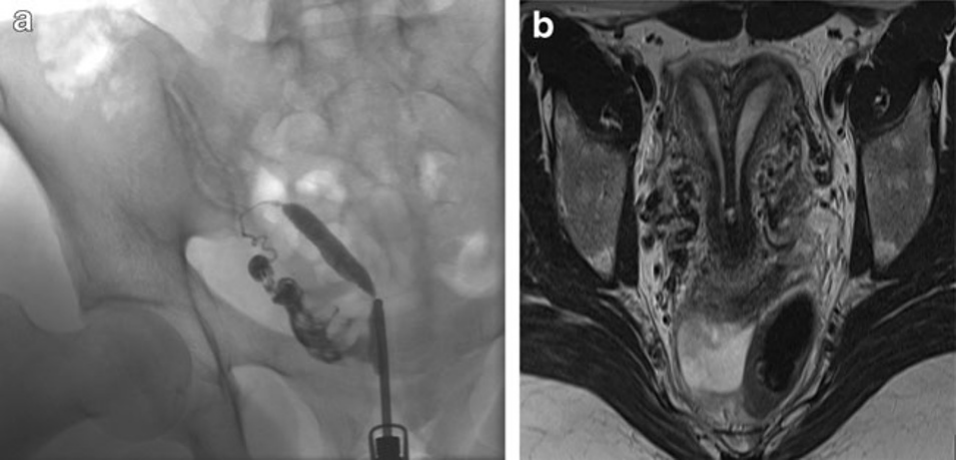
A 31-year-old patient: Suspected uterus unicornis unicollis at HSG (**a**) was identified as bicornis bicollis with MRI (**b**). MRI: T2-weighted, transversal oblique MRI of the pelvis demonstrates two uterine cavities separated by a muscular layer. Uterus didelphys was rated unlikely as no vaginal septum was visible with MRI. Laparascopy is planned

Of these 86 patients, 30 solely uterine pathologies were identified. The most common uterine pathologies were filling defects (n = 15, Figs. 2 and 3) and Müllerian duct anomalies (n = 11), including arcuate uterus, hypoplastic uterus, uterus septus (Fig. 4) and uterus bicornis bicollis (Fig. 5a and b). A few patients (n = 4) had minor abnormalities of the uterine cavity that were described as likely originating from mucosal irregularities (Table 1).

**Fig. 6:**
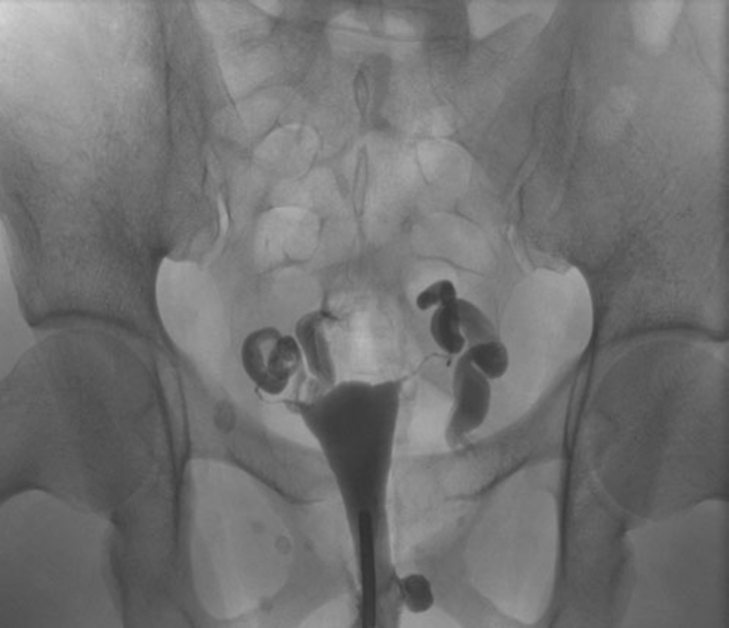
A 37-year-old patient: Dilated fallopian tubes without contrast spillage into the abdominal cavity (bilateral sactosalpinx). Following HSG, an LSC was performed including left-sided salpingectomy and right-sided adhesiolysis because of follicular salpingitis and peritubar adhesions, respectively. The HSC was normal. The patient had no recorded pregnancies to date

**Fig. 7:**
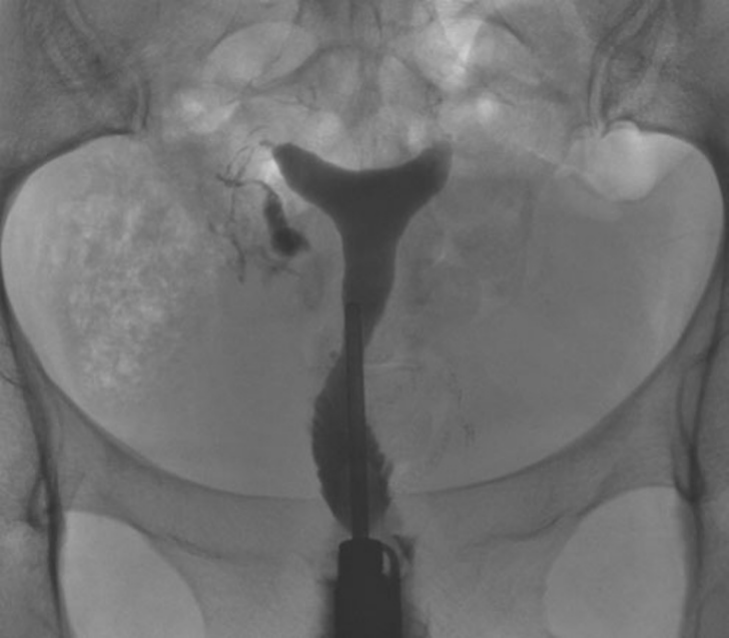
A 38-year-old patient. HSG: Irregular nodular configuration of the right tube (salpingitis isthmica nodosa). The left tube is proximally occluded. Because the patient had decided that she did not want more children, there was no further workup. The HSG was performed to assess the need for anticonception. History: Low anterior rectum resection. Reanastomosis of the right tube. Resection of the left tube. Right tube normal at laparascopy 3 years before HSG

In 47 of the 86 examinations, tubal pathologies were present, and one-sided tubal occlusion (n = 17) was the most common finding. Five of these patients had a history of salpingectomy. Further findings included bilateral tubal occlusion, sactosalpinx (Fig. 6), postinfectious tube abnormalities, including salpingitis isthmica nodosa (Fig. 7), and peritubal adhesions. One patient had several pathological findings. As shown in Table 1, 9 of the 86 HSG examinations demonstrated combined uterine and tubal pathologies. Further diagnostic imaging workups were performed in 25 patients (24 TVUS and 1 MRI of the pelvis; Fig. 5b).

Additionally, 29 laparoscopies (LSC) and 19 hysteroscopies (HSC) were performed on 34 different patients. Three additional pathologies of the uterine cavity (2 small polyps and a single myoma) were identified that were not visible in the HSG. Nine of the 23 one- or two-sided proximal tubal occlusions diagnosed by HSG were normal at laparoscopy, resulting in a false-positive value of 39 %. On the contrary, HSG showed a high negative predictive value (100 %): No additional tube pathologies were identified at LSC if the tube morphology was normal at HSG. In the nine patients mentioned above, LSC (n = 5) and HSC (n = 4) were combined with an intervention (tuboneostomy, myoma resection or one-sided salpingectomy).

A very common procedure following a pathological HSG was insemination because of male subfertility. This procedure was performed as long as one of the fallopian tubes was described as normal and the uterine cavity had no major pathology.

In total, there were 27 pregnancies in 25 of the 86 patients (including 20 patients lost to follow-up, see below) with a pathological condition at HSG. These pregnancies resulted in the birth of 19 healthy children and 8 miscarriages.

Seventeen of the 25 patients only bore a healthy child. These 17 patients had the following HSG results: one-sided tubal occlusion (n = 6), adhesions (n = 4, one of these with uterus arcuatus), filling defects (n = 5), tubal irregularities, possibly postinfectious (n = 1) and sactosalpinx (n = 1).

Two of the 25 women had miscarriages followed by successful pregnancies. Both of these patients showed filling defects, and one patient had additional tube irregularities, which were possibly postinfectious.

Six of the 25 patients had pregnancies that resulted in spontanteous abortions. In these six patients, the HSG showed one-sided tubary occlusion (n = 2), two-sided tubal occlusion (n = 2), adhesions (n = 1) and an arcuate uterus (n = 1).

### Results of further diagnostic workup

Eleven of the 86 patients had no further diagnostic workups. Three of the 86 patients had diagnostic workups following HSG without surgical procedures or further pathologies identified.

Six of the 86 patients that solely had an miscarriage as the outcome showed the following conditions:

- Two-sided tubal occlusion, normal LSC and HSC. Pregnancy after insemination (n = 1).
- One-sided tubal occlusion confirmed at LSC (n = 2), 1/2 with endometriosis at HSC.
- Two-sided tubal occlusion, fundus myoma at HSC not visible at HSG (n = 1).
- Two-sided tubal occlusion, abortion after ovum donation (n = 1). This patient had no LSC because of her relatively high age.
- Uterus arcuatus (ovarian insufficiency) (n = 1).

One of the 86 patients had a miscarriage that was followed by a successful pregnancy. In this patient, the HSG showed a cavity abnormality due to adenomyosis/synechiae. The HSC diagnosis showed multiple scars. An additional myoma that was not visible at HSG was resected (Fig. 3).

Four of the 86 patients had resection of a myoma (n = 3) or a polyp (n = 1) prior to becoming pregnant. Three of these HSG examinations identified filling defects, and one was not visible. One of these four patients had a spontaneous abortion prior to a normal birth. The other three patients gave birth to healthy children.

Forty-one of the 86 patients had no further treatment and no pregnancies. Two of these patients had no wish for further children; the examination was performed to assess the need for anticonception.

For 20 of the 86 (23 %) patients, follow-up information was unavailable. Approximately half of these patients were referred from outside institutions for HSG examinations only, and the remainder were drop-outs from the fertility program.

## Discussion

In our fertility center, HSG is still the most common first-line diagnostic test to evaluate the uterine cavity and tubal patency. HSG is relatively easy to perform and can be completed as an outpatient procedure. Using a good anesthetic protocol with propofol sedation, the procedure is well tolerated by patients. Whereas the administration of Propofol obviously increases patient comfort, the procedure is performed without any sedation in many other institutions, and the advantages should be discussed in light of the additional costs.

As recommended by the ESHRE guidelines [11], we excluded other infertility causes, such as male or ovarian factors, before performing a tubal patency test. According to the ESHRE guidelines, a first-line laparoscopy/hysteroscopy should be performed when tubal, uterine or pelvic pathologies are suspected or detected by TVUS.

In recent years, CAT has become an important screening test that can be used to decide upon further examinations [10]. Hysterosalpingo contrast sonography (HyCoSy) has become an important tool for the diagnosis of uterine or tubal pathologies [12]. According to the literature, this technique has a higher sensitivity than HSG (0.80 versus 0.53) [13], and the specificity (0.84 versus 0.87) is similar for both exams [14].

In our opinion, HyCoSy is more demanding for the examiner than HSG, it is better for assessing the uterine cavity than the fallopian tubes , and it has a longer learning curve. The advantages of hysterosalpingo contrast sonography are that it costs less and causes less pain; therefore, no anesthesia is necessary. Additionally, there is no exposure to radiation.

Whereas HyCoSy and HSG only have diagnostic value, laparoscopy and hysteroscopy also have therapeutic options, including adhesiolysis, excision of endometriotic lesions or resection of intrauterine polyps.

Considering that HSG is far more expensive and invasive (i.e., anesthesia, exposure to radiation) than HyCoSy, our aim is to avoid unnecessary HSG exams. Since 2009, we have routinely used CAT screening to select patients for laparoscopy/hysteroscopy as the first-line exam, and we have set an age limit of 38 years, above which we perform HyCoSy instead of HSG.

During the years of our retrospective study from 2006 to 2010, we chose the diagnostic procedure according to the criteria shown in Table 2. One of the aims of our retrospective study was to improve our decision-making and to avoid unnecessary exams. Of the 411 analysed HSGs, 86 (21 %) demonstrated at least one pathology described as potentially relevant for fertility. This is quite a low detection rate, meaning that we performed four of five HSG procedures in vain. This clearly indicates that the indication for HSG at our institution in recent years was too broad. Furthermore, there was a high false-positive rate for proximal tubal occlusion (39 %) and a high pregnancy rate in patients with pathological HSG, i.e., 25/86 of the total patients and 25/66 of the patients with follow-up.

**Table 2:** Current diagnostic workup for evaluation of tubal and uterine factors in our fertility center

HyCoSy	HSG	Laparoscopy/hysteroscopy
Normal TVUS	Normal TVUS	Relevant pathological TVUS
CAT negative	CAT negative	CAT positive
Planned IVF/ICSI	No history of PID, pelvic surgery or suspected endometriosis	History of PID, pelvic surgery, suspected endometriosis
Planned IUI ♀ ≥ 38 years	Planned IUI ♀ < 38 years	Relevant pathology in HyCoSy or HSG
Planned therapy of ovulatory disorder ♀ ≥ 38 years	Planned therapy of ovulatory disorder ♀ < 38 years	No primary indication for IVF/ICSI

We acknowledge some limitations to our study. First, the retrospective study design might appear to be a limitation of this study; however, all of the images from the examinations had been archived in our PACS, and all pathological cases were re-evaluated. No follow-up information was available for 23 % of the patients with a pathological condition at HSG. It is not known how many of these patients became pregnant. Dropout rates have been reported in the literature to be as high as 50 % before the beginning of therapy. It is likely that patients interrupt their fertility care because of emotional distress and poor prognoses [15]. Furthermore, some follow-ups were not available in our study for patients sent to our center only for tubal assessments by their gynaecologists or family doctors.

Another limitation is that laparoscopic and/or hysteroscopic results were available in only 29 of 411 HSG patients. This means that there was no information about possibly false-negative exams in the patients with normal HSG exams. From the 86 HSG exams with suspected pathologies, 29 patients had laparoscopy/hysteroscopy, and 20 patients were lost to follow-up. Therefore, it is not known how many of these patients became pregnant.

In conclusion, HSG is a safe, low cost and, with the application of propofol sedation, a well-tolerated procedure for tubal assessment, which should be performed at the end of the infertility investigation protocol. The relatively low percentage (21 %) of pathological exams in our population underlines the need for good patient preselection. The high false-positive rate for proximal tubal occlusion (39 %), probably due to tubal spasm, demonstrates the importance of antiperistaltic agents and delayed imaging. Furthermore, we observed a high pregnancy rate (in patients with pathological HSG). Most cases of pregnancy were spontaneous without tubal or uterine surgery. This could mean that the indication for HSG was too broad or that there could even be a therapeutic effect of the HSG procedure, i.e., improved patency of the fallopian tube because of the flushing during the examination [16]. As a result of this data analysis and literature review, the workflow in our own center will be adapted. We will establish hysterosalpingo-contrast sonography (HyCoSy) as a first-line exam for tubal and uterine factors, and improve the patient selection for primary laparoscopy/hysteroscopy using routine screening for CAT.

## Take home points

- With antibiotic prophylaxis, no complications were observed during or following any of the 411 examinations performed at our institution during the study period.
- Low detection rate (21 % pathological exams).
- High false-positive rate for proximal tubal occlusion of 9/23 (39 %), demonstrating the importance of waiting longer after the injection of buscopan to rule out tubal spasm.
- High pregnancy rate in patients with pathological HSG: 25/86 of the total patients and 25/66 of the patients with follow-up. Most pregnancies were spontaneous without tubal or uterine surgery. This could mean that the indication for HSG was too broad or that there could even be a therapeutic effect of the procedure, i.e., that the flushing of the fallopian tubes could improve their patency.
- As a result of this study and the review of the literature, our own center aims to establish hysterosalpingo-contrast sonography (HyCoSy) as a first-line exam to assess tubal and uterine factors and to improve patient selection for primary laparoscopy/hysteroscopy by introducing routine screening for CAT.

## Abbreviations

CAT: Chlamydia antibody titer
ESHRE: European Society of Human Reproduction and Embryology
HSC: Hysteroscopy
HSG: Hysterosalpingography
HyCoSy: Hysterosalpingo contrast sonography
ICSI: Intracytoplasmatic sperm injection
IUI: Intrauterine insemination
IVF: *In vitro* fertilisation
LSC: Laparoscopy
MRI: Magnetic resonance imaging
PACS: Picture Archiving and Communication System
PID: Pelvic inflammatory disease
RIS: Radiology information system
TVUS: Transvaginal ultrasound
